# Effect of the Deposit Temperature of ZnO Doped with Ni by HFCVD

**DOI:** 10.3390/ma16041526

**Published:** 2023-02-11

**Authors:** Delfino R. Gutiérrez, Godofredo García-Salgado, Antonio Coyopol, Enrique Rosendo-Andrés, Román Romano, Crisóforo Morales, Alfredo Benítez, Francisco Severiano, Ana María Herrera, Francisco Ramírez-González

**Affiliations:** 1 IC-CIDS, Benemérita Universidad Autónoma de Puebla, Ed. IC5, Col. San Manuel, Puebla 72570, Mexico; 2 CONACYT-CIO, Lomas del Bosque 115, Col. Lomas del Campestre, León 37150, Mexico; 3 CONACYT-IPN, Av. Insurgentes Sur 1582, Col. Crédito Constructor, Del. Benito Juárez, Ciudad de Mexico 03940, Mexico; 4 Departamento de Investigación en Física, Universidad de Sonora (UNISON), Hermosillo 83190, Mexico; 5 IIIER, Universidad de Ciencias y Artes de Chiapas, Libramiento Norte 1150 Lajas Maciel, Tuxtla Gutiérrez 29039, Mexico

**Keywords:** HFCVD technique, zinc oxide, nickel, dopant, temperature

## Abstract

The effect of the deposit temperature of zinc oxide (ZnO) doped with nickel (Ni) by hot filament chemical vapor deposition (HFCVD) technique is reported in this work. The technique allows depositing ZnO:Ni in short intervals (1 min). A deposit of undoped ZnO is used as a reference sample. The reference sample was deposited at 500 °C. The ZnO:Ni samples were deposited at 500 °C, 400 °C, 350 °C, and 300 °C. The samples were studied using structural, morphological, and optical characterization techniques. The Ni incorporation to the ZnO lattice was verified by the shift of the X-ray diffraction peaks, the Raman peaks, the band gap, and the photoluminescence measurements. It was found that the deposit temperature affects the structural, morphological, and optical properties of the ZnO:Ni samples too. The structure of the ZnO:Ni samples corresponds to the hexagonal structure. Different microstructures shapes such as spheres, sea urchins, and agglomerate were found in samples; their change is attributed to the deposit temperature variation. The intensity of the photoluminescence of the ZnO:Ni improves concerning the ZnO due to the Ni incorporation, but it decreases as the deposit temperature decreases.

## 1. Introduction

Zinc oxide (ZnO) is a semiconductor material with a direct band gap (Eg) of 3.3 eV. This characteristic gives it a wide variety of optoelectronic applications such as light-emitting diodes, solar cells, photodiodes, and transparent conductive oxide films [1,2,3,4]. The scientific interest in ZnO is still increasing because it is abundant, chemically stable, not toxic, has low-cost synthesis, and other properties due to doping and obtention techniques [5,6,7]. Although this material has similar properties to gallium nitride (GaN) [8], the major barrier is the lack of reproducible and low-resistivity p-type ZnO [1]. The experimental conditions allow obtaining ZnO as a thin film or nanostructured material. Nanostructured ZnO opens a large field of application since the nanostructured material presents new and different optical and electrical properties. Moreover, these nanostructures can be grown in diverse morphologies and shapes such as nanotubes, nanowires, nanodiscs, etc [9,10,11]. The control of ZnO properties is critical for novel applications. By establishing the conditions of synthesis, the properties of the material can be controlled. Under this principle, a better understanding of the synthesis of microstructures with different morphologies is important and necessary for ZnO applications.

Different techniques have been used to deposit ZnO such as chemical vapor deposition (CVD) [12], spray-pyrolisis (SP) [13], magnetron sputtering [14], pulsed laser deposition (PLD) [15], sol-gel [16] and physical vapor deposition [17]. The hot filament chemical deposition (HFVCD) technique allows the deposit of semiconductor materials with high deposition rates, low operation cost, and good control of the experimental conditions. HFCVD technique has been used to deposit a wide variety of materials including diamond [18], aluminum, silicon, and titanium nitride [19], gallium nitride [20], nanostructures of silicon-rich [21], nanocrystalline silicon [22], graphene [23], molybdenum selenide [24], silicon carbide [25], and zinc sulfide [26], among others.

A difference between CVD and HFCVD is that the sources are solid (pellets), and they can be brought to the vapor phase in a short time by the heating method; the control of the temperature deposit and the rate flow of the reactants influence the structure, morphology, electrical, and optical properties of the deposited ZnO. On the other hand, the ZnO doping with transition metals also modifies their properties, which is suitable for various application fields such as optoelectronics, gas sensing, and photocatalysis. Some of the transition metals used for ZnO doping include Ti, Mn, Co, Sn, Fe, and Ni. Then, the technique and synthesis conditions influence the ZnO doping, altering the ZnO properties. In this work, a homemade hot filament chemical deposition (HFVCD) system was used to deposit ZnO:Ni microstructures. The incorporation of the Ni into the ZnO and the effect of the deposit temperature (on the structural, morphological, and optical properties of the ZnO:Ni) are presented. Our particular interest is determining the minimum temperature to incorporate Ni in the ZnO lattice by HFCVD.

## 2. Materials and Methods

### 2.1. HFCVD Technique

**Figure 1:**
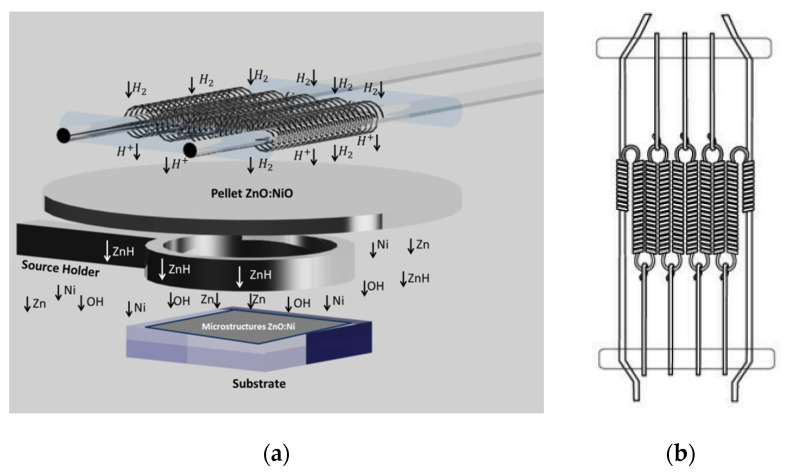
(**a**) Schematic representation of the experimental arrangement. The flow through the HFCVD reactor is vertical, perpendicular to the filaments. It is possible to move the substrate holder to change the deposit temperature. (**b**) Schematic representation of the filament arrangement, the source is under it. H_2_ flows through the filament to the source, the substrate holder is under the source. The position of the substrate holder is settled to control the deposit temperature.

The deposit of semiconductor materials using the hot filament chemical vapor deposition (HFCVD) technique is widely reported [21,23,24,25,26,27]. The material of the filament, used in the experimental arrangement, is selected to reach temperatures of about 2000 °C, usually tungsten, molybdenum, or tantalum. The temperature that the filament reaches and the deposit temperature could be different. The deposit temperature is settled by controlling the distance between the filament and the deposit area. Reactants in the gas phase are generated by attacking a solid source with atomic hydrogen (H). Figure 1a shows a schematic representation of the experimental arrangement used in this work. When molecular hydrogen (H_2_) passes through the hot filament, the H_2_ bonds dis in H radicals, which are highly reactive. Then, dissociated H radicals attack the solid source, forming the precursors of the semiconductor materials in the vapor phase.

In our system, the flow is perpendicular to a filament arrangement. Figure 1b shows a schematic representation of the filament arrangement. The source is between the filament and the substrate holder. The distance of the substrate holder is controlled to set the deposit temperature.

In the HFCVD technique, when the source and the atomic hydrogen are in contact, the reaction starts. The hydrogen carries atoms from the source to the substrate, which is at a lower temperature, and the carried atoms start nuclei forming films and nanostructures. In our work, ZnO pellets are used as a source, the oxygen ions are removed from the lattice by reduction, due to the temperature and when the concentration of vacancies reaches a certain critical value, they are annihilated by rearrangement of the lattice with the eventual formation of metal nuclei. Metal nuclei eventually evaporate to form precursors in the gas phase, which are carried by the flow to the substrate.

### 2.2. Samples Preparation and Deposit

Pellets of ZnO and ZnO with NiO (ZnO:Ni) were made to be used as a solid source in the HFCVD reactor. Powders of ZnO (Mallinckrodt Chemical CAS 1314-13-2, St. Louis, MO, USA) and NiO (ALDRICH CAS 1313-99-1, St. Louis, MO, USA) were used to form the solid source, the powders were compressed with a pressure of 600 kgf/cm. Both kinds of pellets, ZnO and ZnO:Ni, were made under the same pressure, size, and weight (500 mg) conditions. The ZnO:Ni pellets were made using a mixture of ZnO and NiO powders with a mass ratio of 1:1.

Samples of p-type crystalline silicon (100), with 5–10 Ω⋅cm resistivity, were used as substrates. The substrates were cleaned in an ultrasonic bath with xylene, acetone, and methanol for 10 min in each solution. Then, the substrates were immersed in a 10% aqueous solution of hydrofluoric acid (HF) for 2 min. The deposit was made on the specular face of the substrates using a chromatographic H_2_ (supplied by INFRA) flow of 50 sccm through the reactor chamber, while the filament was hot at 2000 °C. The distance between the source and the filament was 2 mm and it was kept constant in all the deposits. On the other hand, the distance between the source and the substrate was 1 mm, 2 mm, 3 mm, and 3.5 mm; these distances correspond, in our system, to a deposition temperature (substrate temperature) of 500 °C, 400 °C, 350 °C, and 300 °C, respectively. A detailed description of the used HFCVD can be found in the reference [28]. The deposit time was 1 min for all the samples. The samples are listed in Table 1.

The structure of the samples was studied by X-ray diffraction (XRD) measurements, which were carried out on a Bruker AXS D8 Discovery diffractometer. Cop radiation with a wavelength of 1.54 Å, (Billerica, MA, USA) was used. The photoluminescence (PL) characterization was made using a Fluoro-Max 3 system (HORIBA, Ltd., Kyoto, Japan), with an excitation wavelength of 330 nm. Uv-vis measurements were performed in a Cary 5000 (Agilent-Varian, Australia) spectrophotometer with the diffuse reflectance accessory. The Raman spectra were obtained with a Micro-Raman Horiba LabRAM-HR (HORIBA, Les Ulis, France) instrument, with a laser excitation wavelength of 632.8 nm. The morphological characterization of the samples was made using an AURIGA 3916 –FESEM (Zeiss, Jena, Germany) scanning electron microscope.

## 3. Results and Discussion

### 3.1. Structural Characterization

#### 3.1.1. X-ray Diffraction

**Figure 2:**
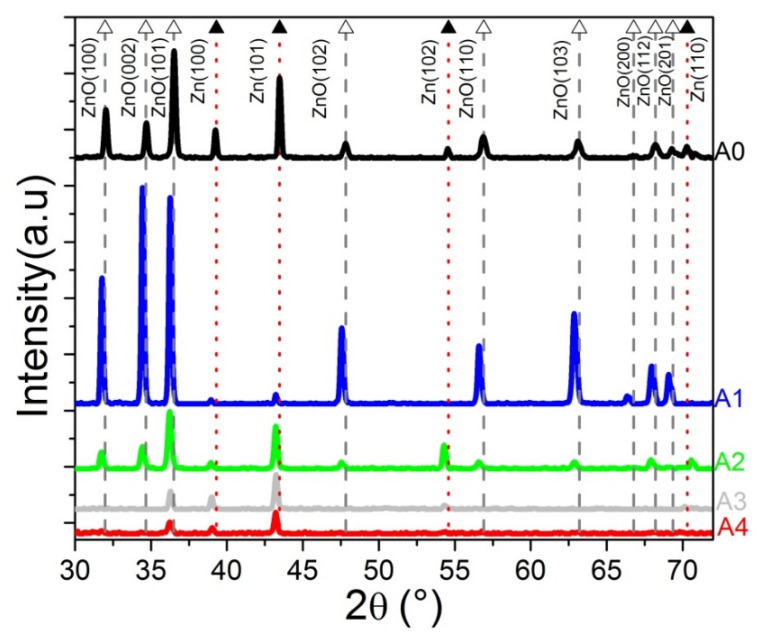
XRD diffraction patterns. All the samples were deposited by HFCVD for 1 min using a solid source. The source of the A0 sample was ZnO without NiO. The deposit temperatures of the ZnO:Ni samples (source of ZnO with NiO 1:1) were 500 °C, 400 °C, 350 °C, and 300 °C labeled as A1, A2, A3, and A4, respectively. The diffraction peaks shift to the left, taking A0 as a reference. The peak shift is attributed to Ni atoms incorporated in the ZnO lattice.

The crystal structure of the samples was analyzed by XRD. The XRD diffractograms are shown in Figure 2. The sample A0, deposited at 500 °C, corresponds to the sample without doping with NiO, and it is used as a reference in our analysis. The diffraction peaks of A0 are located at 31.83°, 34.55°, 36.74°, 47.75°, 56.76°, 63.09°, 66.77°, 67.93°, and 69.25°, which correspond to the planes (100), (002), (101), (102), (110), (103), (200), (112), and (201) of the ZnO hexagonal structure (ICDD #00-036-1451, denoted with empty triangles in Figure 2). The localization of the ZnO peaks is indicated with a gray dotted vertical line. On the other hand, the peaks located at 39.17°, 43.25°, 54.47°, and 70.79° correspond to the planes (100), (101), (102), and (110) of the metallic Zn in the hexagonal phase (ICDD # 00-001-1244, denoted with solid triangles in Figure 2). A vertical dotted red line indicates the localization of the Zn peaks. The ZnO:Ni samples were deposited at 500 °C, 400 °C, 350 °C, and 300 °C labeled as A1, A2, A3, and A4, respectively. No diffraction peaks related to Ni or NiO were found. All the peaks of ZnO:Ni present a shift to the left, in comparison to the diffraction peak of A0. The effect of the incorporation of the Ni atoms in the ZnO is an increment of the ZnO lattice size; this is caused by a compression-type micro-stress attributed to the Ni atoms that act as a substitution of the Zn atoms within the ZnO structure [29] due to the difference in ionic radii between the ZnO and the Ni dopant ion.

Both A0 and A1 were deposited at 500 °C, but ZnO diffraction peaks were more intense in A1 than in A0, and the peaks corresponding to Zn were barely noticeable (A1). The intensity of the diffraction peaks of metallic Zn at 43.3°, 54.5°, and 70.8° increase concerning the ZnO peaks as the deposit temperature decreases to such a degree that in the samples A3 and A4 present, only one diffraction peak at 47.8° related to the ZnO structure. The lattice parameters a and c were calculated using Equation (1).

(1) $$\frac{1}{d_{\left(hkl\right)}^{2}}=\frac{4}{3a^{2}}\left(h^{2}+k^{2}+hk\right)+\frac{l^{2}}{c^{2}}$$

where the interplanar spacing $d_{hkl}$ is related to the diffraction angle θ. The angle θ is obtained from the XRD pattern, where $\frac{1}{d_{hkl}}=\frac{2sin\theta }{\lambda }$. The lattice parameters a and c were calculated using the XRD peaks at 31.85° (100) and 34.45° (002) for ZnO, and 36.34° (002) and 39.31° (100) for Zn [30]. Table 2 shows the calculated lattice constants a and c from the XRD peaks related to ZnO and Zn. A slight increase in the lattice parameter a can be seen for the ZnO; the same situation is also observed for lattice parameter c of the Zn. This is attributed to the ionic radius difference between Ni^2+^ and Zn^2+^. Therefore, the Ni atom is introduced as a dopant.

#### 3.1.2. Micro-Raman Spectroscopy

**Figure 3:**
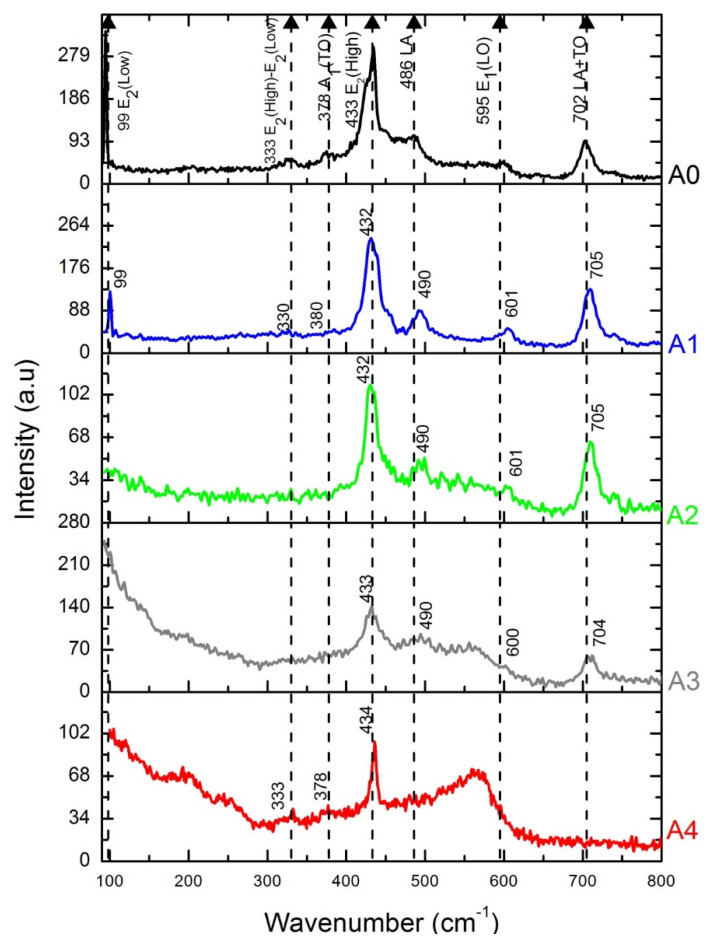
Raman spectra. A0 sample is the ZnO deposited without NiO at 500 °C. The deposit temperatures of the ZnO:Ni samples were 500 °C, 400 °C, 350 °C, and 300 °C labeled as A1, A2, A3, and A4, respectively. The vibrational modes E2 (low, ~101 cm^−1^) and E2 (high, ~430 cm^−1^) are related to the hexagonal structure of the ZnO. The vibrations modes ~490 cm^−1^ and ~708 cm^−1^ are related to defects [35].

Raman scattering spectrum is used to determine the incorporation of the Ni ions into the ZnO lattice. Raman scattering spectra at room temperature ranging from 100 to 1000 cm^−1^ are shown in Figure 3. The more intense peak at 430 cm^−1^ is related to the vibrational mode E2 (high) of the hexagonal structure of the ZnO [31]. The stability of the hexagonal structure upon Ni doping is ascribed to the comparable Ni and Zn ionic radii, 0.69 Å and 0.74 Å for Ni^2+^ and Zn^2+^, respectively [32,33]. The shift of the peak from 430 cm^−1^ in A0, to 428 cm^−1^ in A1, is related to the Ni doping. The peak at 101 cm^−1^ corresponds to the E2 (low) mode of the hexagonal ZnO structure [34]. The peaks at 604 correspond to the longitudinal-optical mode. E1(LO). The vibrations around 490 cm^−1^ and at 708 cm^−1^ were reported in the reference [35]; these can be related to defects such as oxygen vacancies, zinc interstitials, and antisites of oxygen.

#### 3.1.3. XPS

**Figure 4:**
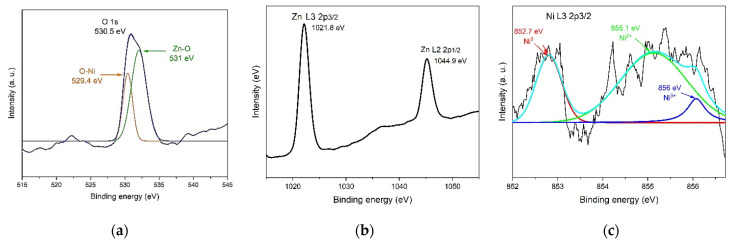
XPS survey spectrum of the sample A1. (**a**) O 1s, (**b**) Zn L3 2p3/2 and L2 2p1/2, (**c**) Ni L3 2p3/2.

The presence of Ni into the ZnO lattice was investigated by X-rays photoelectron spectroscopy (XPS). Figure 4 shows the XPS survey spectra of the sample A1 for O, Zn, and Ni. The binding energy ~530 eV in regard to 1s O is shown in Figure 4a; the subpeak ~529.4 eV is related to oxygen binding to Ni, and the subpeak ~531 eV is attributed to Zn–O binding. The binding energies ~1021 eV and ~1044 eV correspond to Zn energy states of L3 2p3/2 and L2 2p1/2, respectively, shown in Figure 4b. The energy band concerning to the Ni L3 2p3/2 spectrum was deconvoluted to observe the Ni binding energies ~852 eV, ~855 eV, and ~856 eV which coincided with of Ni^0^, Ni^2+^, and Ni^3+^ energy states [36,37], respectively shown in Figure 4c. The intensity of the Ni energy band was weak, and it was not appreciable in the other samples. The Ni incorporated into the ZnO lattice decreases as the deposit temperature decreased. This result agrees with the XRD and Raman characterizations.

### 3.2. Morphological Characterization

#### Scanning Electron Microscopy

**Figure 5:**
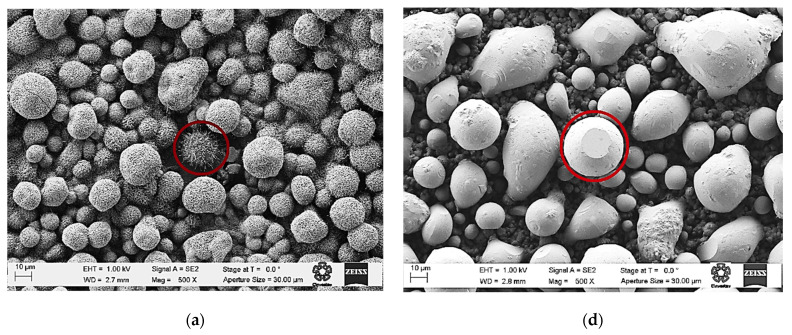

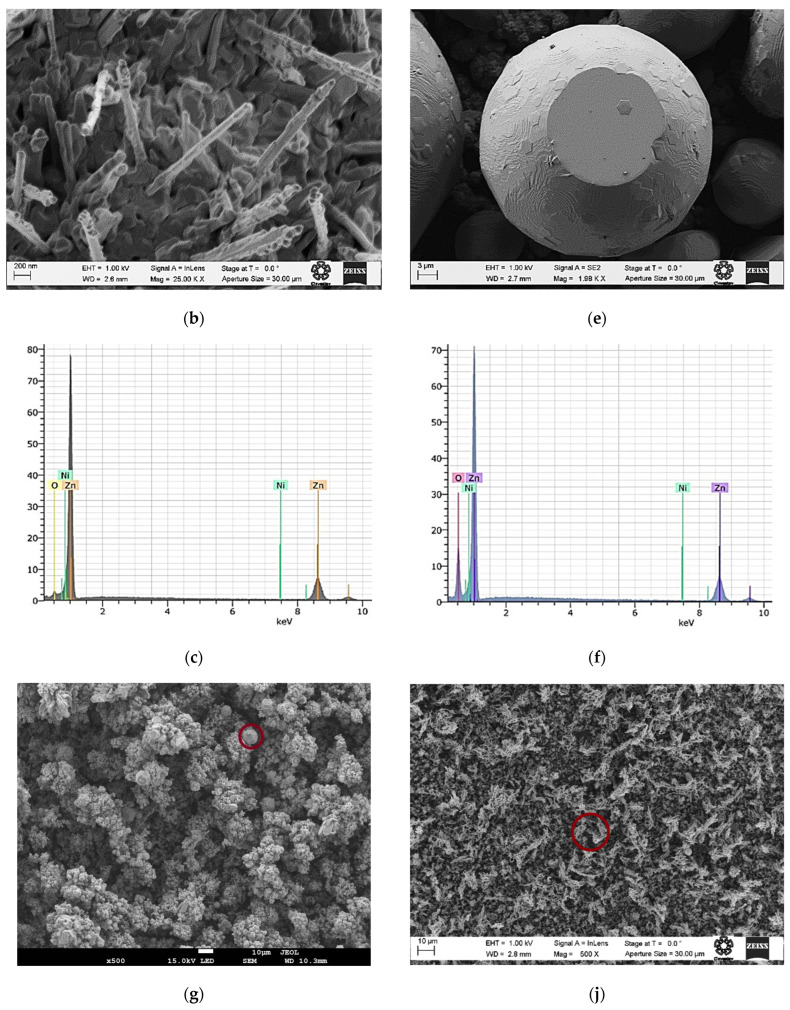

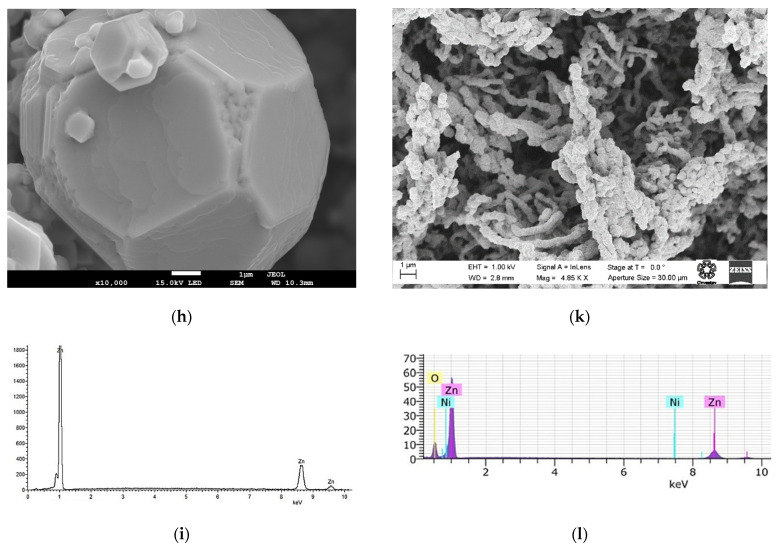
SEM micrographs of samples A1 (**a**–**c**), A2 (**d**–**f**), A3 (**g**–**i**), and A4 (**j**–**l**) are arranged in columns. In each micrograph set the image at the top corresponds to a larger area, a magnification of a particular structure is presented in the middle, and an elemental composition analysis (EDS) is at the bottom of each column.

The morphology was analyzed by scanning electron microscopy (SEM). The micrographs of samples A1, A2, A3, and A4 are arranged in columns and shown in Figure 5. The presence of different microstructures, whose morphology depends on the deposit temperature, is observable. In each column, the micrograph in the first row corresponds to a larger area, then, a magnification of a particular structure is presented. Finally, an elemental composition analysis (EDS) of the observed structure in the magnifications is presented in the last row. The average atomic percentage of the EDS is shown in Table 3. The measurements were taken in three different places on each sample. The amount of Ni is similar in A1, A2, and A3 samples, but it is incorporated in the ZnO lattice at 500 °C, as observed in XPS. The EDS detection in A3 and A4 is related to Ni, which is not doping the ZnO.

Figure 5a corresponds to sample A1, where sea urchin shape structures, with wires on their surfaces, are observed. A magnification of a sea urchin shape structure is shown in Figure 5b. Using the scale in the image, ~1 μm wires length and ~100 nm wires diameter were estimated. An elemental composition analysis (EDS) of the sea urchin shape structure is presented in Figure 5c. According to XPS, there was Ni incorporation into the ZnO lattice, but it was low. The sample has good crystallinity quality related to XRD. We propose that the microstructures grow from Zn drops, which were previously formed on the substrate when the surface reached 500 °C; the melting point of the Zn is 420 °C. Then, a nucleation stage occured, where the O atoms bound with the Zn drops, forming Zn and ZnO Core-Shell structures, where the Ni atoms acted as a catalyst, favoring the growth.

The micrograph of sample A2 shows the presence of elongated quasi-spheres microstructures, presented in Figure 5d, with different size, and diameters between 2 and 40 μm (estimated using the micrograph). The elongated structures could be formed by the nucleation of two or more nearby Zn drops forming quasi-spheres due to the temperature deposit. Then, the O atoms bound with the structures, forming Zn and ZnO Core-Shell structures. In this case, the amount of Ni was lower than A1, related to XRD, limiting the growth of the wires. Figure 5e shows a magnification of a quasi-sphere microstructure with a flat shape end. Truncations on the surface of the microstructures and flat plates have been associated with the hexagonal ZnO structure [28]. Figure 5f show an EDS of the quasi-sphere microstructure. The crystallinity quality was lower than A1, related to XRD, meanwhile, the XPS signal, to detect the Ni, was too low to be reliable, but the effect of the Ni was observable in the optical characterizations (photoluminescence and diffuse reflectance).

Figure 5g shows the micrograph of the A3 sample, where agglomerates with a rough surface are observed growly arbitrarily. In the magnification of Figure 5e, a structure with smooth hexagonal faces was observed; an EDS of the structure is presented in Figure 5i. There was no Ni present in this sample; according to XRD and EDS, the material was almost Zn with a low percentage of ZnO. We propose that ZnO droplets start to nuclei, forming agglomerates. These agglomerates are the precursors of the morphology obtained when the deposit temperature was increased to 400 (sample A2).

The A4 sample morphology is shown in Figure 5j. The surface had a high roughness and nearly a porous surface. Figure 5k is a magnification, where an interconnected formation is observed. In the background, many spheres are observed, with diameters between 1 μm and 4 μm. From the background, some agglomerates are interconnected in one direction, forming fibers with diameters of ~0.5 μm. The corresponding EDS is presented in Figure 5l. According to XRD and EDS, Ni was not present in the sample. It is proposed that when the temperature is below 400 °C, Zn droplets start to nuclei, forming the agglomerates, which are interconnected to form fibers at 300 °C. Then, when the deposit temperature is 350 °C, the agglomerations growth, and mounds form. At 400 °C, the mounds form smooth structures where Ni is incorporated. Finally, at 500 °C, the Ni favors the growth of needles on the surface of the structures.

### 3.3. Optical Characterization

#### 3.3.1. UV-Vis Spectroscopy

**Figure 6:**
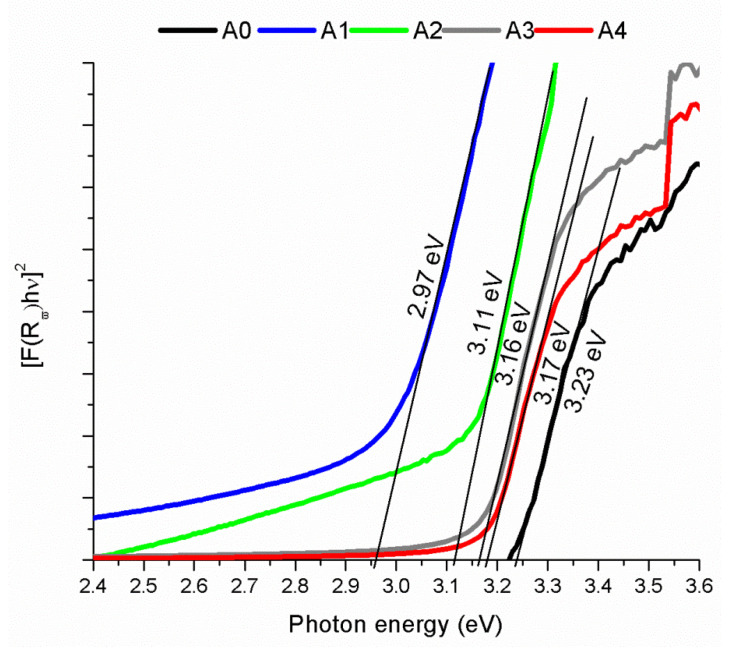
UV-DRS. The Kubelka–Munk approximation was used to estimate the Eg. A0 is the reference sample deposited without Ni at 500 °C. The samples A1, A2, A3, and A4 were deposited with ZnO:Ni at 500 °C, 400 °C, 350 °C, and 300 °C, respectively. The change of the band gap Eg is attributed to the incorporation of the Ni in correlation with the deposit temperature.

Figure 6 shows the absorption spectra of the samples obtained by diffuse reflectance (UV-DRS), where the band gap (Eg) in the range of 2.97 eV and 3.23 eV was calculated using the Kubelka–Munk approximation [38]. Band gap (Eg)values between 2.97 eV and 3.23 eV were obtained. The sample A0 (whose deposit source is only ZnO) presented an Eg of 3.23 eV, typical for ZnO. For the samples deposited with ZnO:Ni, a shift towards lower energies can be observed. The lowest Eg energy was estimated in A1 (2.97 eV), then as the deposit temperature decreased, the Eg tended to the ZnO value (3.23 eV). The estimated Eg of the samples A3 and A4 was around the same. The change of the Eg is attributed to the incorporation of the Ni in correlation with the deposit temperature.

The redshifts of the edge of the absorption band of ZnO doped with Ni was observed by Elilarassi and Chandrasekaran et al. [30] in the exchange interactions between the band sp of electrons in ZnO and electron-d of ions of Ni^2+^. This confirms that Ni is acting as a dopant in the structure. The reduction in the bandgap is the effect of the temperature deposit of the samples.

#### 3.3.2. Photoluminescence

**Figure 7:**
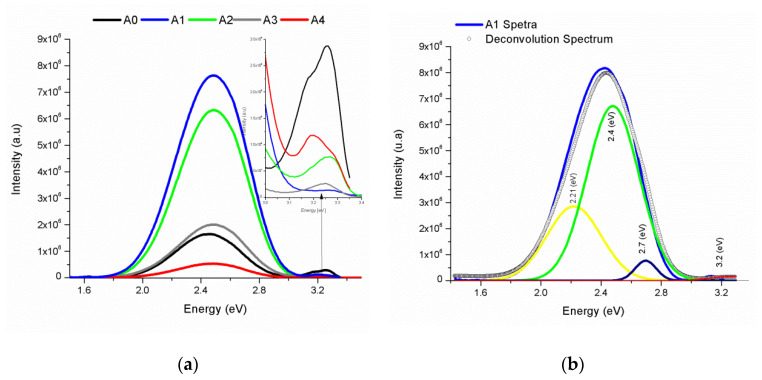
Photoluminescence spectra. The A0 spectrum is used as a reference, this sample was deposited without Ni. (**a**) PL spectra of the samples A1, A2, A3, and A4, deposited with ZnO:Ni at 500 °C, 400 °C, 350 °C, and 300 °C, respectively. The intensity of the emission peak ~2.48 eV of A1 is around four times the intensity peak of A0. The PL intensity (A2, A3, and A4) was reduced as the deposit temperature decreased. The effect of the temperature on the NBE band ~3.2 eV (upper right corner) is an intensity increment, lower in A1. (**b**) Deconvolution of the A1 PL spectrum, the peaks around 2.21 eV, 2.4 eV, 2.7 eV, and 3.2 eV are associated with different types of defects.

Photoluminescence (PL) at room temperature was measured to study the effect of the inclusion of Ni atoms in the ZnO, and the effect of the deposit temperature on the PL emission. The PL spectrum of the reference sample A0, deposited without Ni, and the PL spectra of the samples A1, A2, A3, and A4, deposited with ZnO:Ni, are shown in Figure 7a. Two PL emission bands are presented in the spectra of the Figure 7a, a broadband emission centered at 500 nm (2.48 eV), and an emission band centered at 380 nm (3.2 eV). Regarding the A0 spectrum, the emission peak ~2.48 eV of A1 increased its PL intensity around four times. This effect is attributed to the Ni atoms incorporated in the ZnO lattice. It was observed that the emission peak decreased as the deposit temperature decreased (A2, A3, and A4). The peak of the emission band ~3.2 eV originated at the near band edge (NBE) transition in the UV for ZnO, and it is related to the free excitons recombination [33,39]. The intensity of the NBE band was reduced in the A1 spectrum with respect to the A0 spectrum (a zoom of the NBE band is shown in the upper right corner.). The intensity of the NBE band increased as the deposit temperature decreased. Deconvolution of the A1 spectrum is presented in Figure 7b. The peaks around 2.21 eV, 2.4 eV, 2.7 eV, and 3.2 eV are associated with different types of defects such as Zn_i_, O_i_, V_Zn_, V_O_, and O_Zn_ [40]: Oxygen vacancies (V_O_), zinc vacancies (V_Zn_), interstitial zinc (Zn_i_), interstitial oxygen (O_i_), and oxygen antisites (O_Zn_) or some transition metals [41,42,43].

The Green emission in undoped ZnO has been associated with the presence of V_O_ and V_zn_ [44,45]. Both introduce deep acceptors in the band gap. Green luminescence is caused by the electronic transition between shallow donors and deep acceptors. It is widely known that V_O_ exists when the ZnO material is Zn rich. In our case, the samples were Zn rich, which prompted V_O_ defects. The reduction in the band gap observed by Uv-Vis (Figure 6) due to the introduction of Ni atoms could mean that the impurity introduces states near the conduction band in the ZnO. The transition between those states and V_O_ (deep acceptor states) might be the origin of the Green luminescence. Additionally, a blue luminescence band emerged around 450 nm (2.21 eV) in the photoluminescence spectrum of ZnO. Recently, Zeng et al. [46] and Cao et al. [47] obtained visible dichromatic photoluminescence emitting in the blue/violet and green region by introducing Zn_i_ defects in the structure ZnO.

## 4. Conclusions

ZnO:Ni samples were successfully deposited by hot filament chemical vapor deposition (HFCVD) at different temperatures. A ZnO deposit (without Ni) is used as a reference sample. The Ni incorporation to the ZnO lattice was verified by the XRD, Raman, XPS, UV-Vis, and PL characterizations. The crystalline structure of the ZnO:Ni samples corresponds to the hexagonal ZnO structure, where neither Ni nor NiO structures were found. The Ni improved the PL intensity at 500 °C, with respect to the reference sample, but the PL intensity decreased as the deposit temperature decreased; the Ni incorporation into the ZnO lattice was corroborated by XPS. On the other hand, the NBR band increased as the deposit temperature decreased, the NBR band almost disappeared when the deposit temperature was at 500 °C. In concordance, the calculated band gap shifted to lower energy (2.97 eV, ZnO:Ni deposited at 500 °C) and it trended toward the undoped ZnO (3.23 eV) as the deposit temperature decreased. The effect of the deposit temperature was observed in all the results, but it is on the morphology where the effect is more appreciable. Different ZnO microstructures were obtained at different deposit temperatures. The trend of the morphology is from agglomerates, growing in the 1D direction, to form spheres, and the change was achieved without thermal treatment.

## Figures and Tables

**Table 1:** Samples labels and deposit temperatures. All the samples were deposited for 1 min using an H_2_ flow of 50 sccm. The source-filament distance was 2 mm, constant in all the deposits. The variation of the filament-substrate distance produces a different substrate temperature (deposit temperature) [28].

Sample Label	ZnO:NiO Weight Ratio	Substrate Temperature (°C)	Source-Filament Distance (mm)
A0	1:0	500	1
A1	1:1	500	1
A2	1:1	400	2
A3	1:1	350	3
A4	1:1	300	3.5

**Table 2:** Calculated lattice parameters. The lattice parameters a and c were calculated using the Equation (1) and the XRD peaks at 31.85° (100) and 34.45° (002) for ZnO, and at 36.34°(002) and 39.31° (100) for Zn. The effect is due to the change in the deposit temperature.

Label	ZnO Lattice Parameters (Å)		Zn Lattice Parameters (Å)	
	a	c	a	c
A0	3.248	5.207	2.67	4.965
A1	3.251	5.206	2.67	4.966
A2	3.250	5.206	2.67	4.966
A3	-	-	2.66	4.965
A4	-	-	2.66	4.970

**Table 3:** Average of the atomic percentage of the elemental composition of the samples. The atomic percentage of Ni decreases as the deposit temperature changes.

	Average Atomic Percentage of Elements		
Sample Label	Zn	O	Ni
A1	80	19.66	0.29
A2	80.11	19.66	0.23
A3	80.13	19.66	0.21
A4	80.21	19.66	0.13

## Data Availability

The raw data required to reproduce these findings are available to download from “[https://data.mendeley.com/datasets/bptvhg5zk3/1] 9 December 2021”. The processed data required to reproduce these findings are available to download from “[https://data.mendeley.com/datasets/bptvhg5zk3/1] 9 December 2021”.
